# Supplementary material for: Disentangling metabolic functions of bacteria in the honey bee gut
Source: PLoS Biol. 2017 Dec 12;15(12):e2003467. doi: 10.1371/journal.pbio.2003467 (PMC5726620; doi:10.1371/journal.pbio.2003467)
Supplement: S8 Data — (ZIP) [file pbio.2003467.s008.zip › Z-score_plots/784 microbial product 11-O-Demethyl-17-O-deacetylvindoline.pdf]

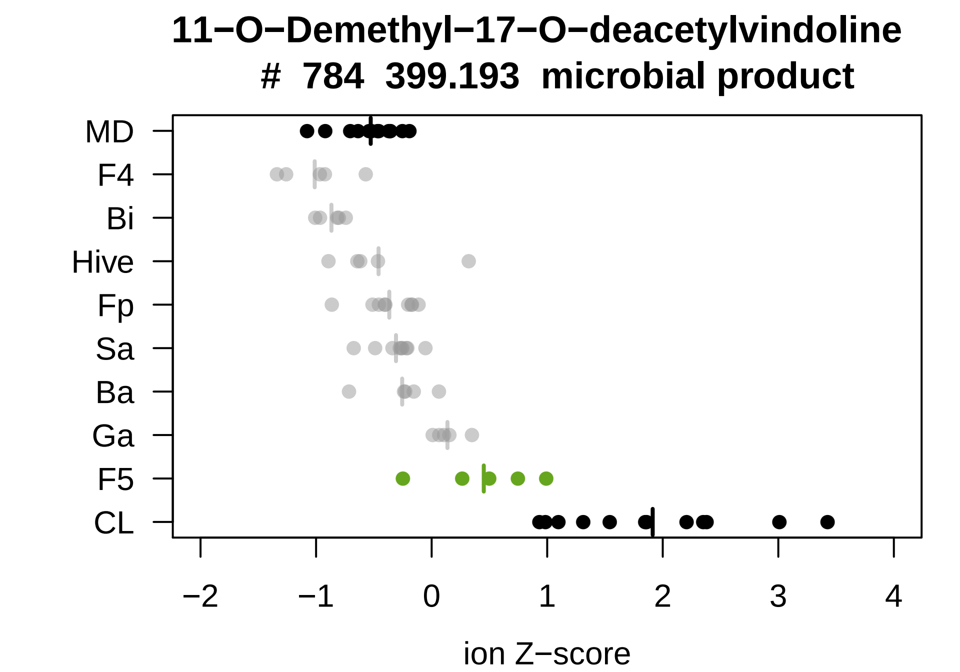
11−O−Demethyl−17−O−deacetylvindoline
784 399.193 microbial product
MD
F4
Bi
Hive
Fp
Sa
Ba
Ga
F5
CL
−2
−1
0
1
2
3
4
ion Z−score
